# Supplementary material for: Multi-site Assessment of Methods for Cell Preservation Upstream of Single Cell RNA Sequencing
Source: bioRxiv. 2025 Oct 24:2025.10.24.684427. Preprint. [Version 1] doi: 10.1101/2025.10.24.684427 (PMC12633272; doi:10.1101/2025.10.24.684427)
Supplement: 1 [file NIHPP2025.10.24.684427V1-supplement-1.pdf]

bioRxiv preprint doi: https://doi.org/10.1101/2025.10.24.684427; this version posted October 24, 2025. The copyright holder for this preprint (which was not certified by peer review) is the author/funder, who has granted bioRxiv a license to display the preprint in perpetuity. It is made available under aCC-BY 4.0 International license.

Supplemental Materials

Figure S1 - Container-based Rstudio analysis environment

Our study involved multiple collaborators at different institutions with various computer infrastructures and required a computational solution to provide a consistent analysis environment. A) A Docker Rstudio image containing all required software packages was prepared and deposited in Docker Hub (docker://alemenze/abrfseurat). B) This container can be executed using Docker, Singularity, or Apptainer in any computational environment. Given the scale of the data in this study, the most common environment used was a Linux-based high performance computer (HPC) cluster. C) After launching the R environment, contributors can interact with the software from the command-line or by using the browser-based Rstudio interface.

Figure S2 - Cluster-level debris exclusion

Low quality leukocyte clusters with ambiguous cell identity were excluded from analysis prior to downsampling. A) Leukocytes from 10x 3pGEX or 10x FLEX and B) Leukocytes prepared with HIVE technology. Left panels are cluster assignments used to evaluate real vs debris clusters. Middle panels are the same UMAPs but colored according to real cell flag (purple) or debris (grey). Right panels are violin plots of l2.n_count and l2.n_feature statistics stratified by debris assignment using the same color scheme as the adjacent UMAPs plots. Debris clusters are generally in the center of the UMAP and have poor quality control statistics compared to real cells.

Figure S3 - Sequencing summary

Bar charts of different sequencing parameters for the 24 samples considered in this study. In each panel, colors are according to technology and storage parameters. The leukocytes are the 20 bars to the left, PBMCs are the 4 bars to the right. A) Overall depth of sequencing in millions of reads. B) Cell counts per sample after debris exclusion. C) Sequencing depth in reads per cell prior to downsampling input FASTQ files. D) Sequencing depth in reads per cell after downsampling FASTQ files to 25,000 reads per cell. These downsampled FASTQ files were then used as input in all analyses.

Figure S4 - Quality control statistics for Leukocyte and PBMC samples stratified by general cell type

Violin plots presenting the log2 transformed gene count (l2.n_feature), read counts (l2.n_counts) and the percent mitochondrial reads (mitoRatio) for filtered and downsampled leukocyte (A) and PBMC (B) samples. The leukocyte site-level replicates have been merged for this presentation and the values for each general cell type are plotted separately. Mitochondrial read percentages are relatively low in the 10x FLEX samples because probes interrogating these genes are excluded from the platform. Violins are colored according to technology. Dashed reference lines are provide at 6.6 (100) and 9 (500) for gene counts in l2.n_feature panels, 10 (1000) and 12.3

bioRxiv preprint doi: https://doi.org/10.1101/2025.10.24.684427; this version posted October 24, 2025. The copyright holder for this preprint (which was not certified by peer review) is the author/funder, who has granted bioRxiv a license to display the preprint in perpetuity. It is made available under aCC-BY 4.0 International license.

(5000) for read counts in l2.n_count panels and 0.1 (10%) for percent mitochondrial reads in mitoRatio panels.

Figure S5 - Post-integration UMAP plots of each sample

Following integration of leukocyte (A) and PBMC (B) data, UMAP plots show similar distributions of cells in these two dimensional representations indicating successful integration of data from different technologies. UMAP plots are colored according to cluster.

Figure S6 - FACS tSNE plot

The CD45+ leukocyte population as characterized by flow cytometry using a 21-color panel of surface marker antibodies (See Table S1).

Figure S7 - Differential expression analysis of HIVE 4 weeks vs HIVE 1 day leukocyte samples

Volcano plot of differential expression results between the HIVE 28 day storage A replicates and the HIVE 1 day A replicate. Genes with the largest magnitude positive and negative log2 fold changes are labeled. No genes exceed the abs(log2 Fold Change) > 1, FDR < 0.05 threshold for differential expression.

Table S1: Flow Cytometry Antibodies

SPECIFICITY	FLUOROCHROME	CLONE	CATALOG #	VENDOR
CD45RA	BUV395	5H9	740315	BD
CD16	BUV496	3G8	564653	BD
CD56	BUV737	NCAM16.2	564447	BD
CD8	BUV805	SK1	612889	BD
CD197 (CCR7)	BV421	G043H7	353208	BioLegend
CD123	Super Bright 436	6H6	62-1239-42	ThermoFisher
CD11c	eFluor 450	3.9	48-0116-42	ThermoFisher

bioRxiv preprint doi: https://doi.org/10.1101/2025.10.24.684427; this version posted October 24, 2025. The copyright holder for this preprint (which was not certified by peer review) is the author/funder, who has granted bioRxiv a license to display the preprint in perpetuity. It is made available under aCC-BY 4.0 International license.

IgD	BV480	IA6-2	566138	BD
CD3	BV510	SK7	344828	BioLegend
CD28	BV650	CD28.2	302946	BioLegend
CD14	Spark Blue 550	63D3	367148	BioLegend
CD45	PerCP	HI30	368506	BioLegend
TCRγδ	PerCP-eFluor 710	B1.1	46-9959-42	ThermoFisher
Siglec-8	PE	7C9	347104	BioLegend
CD4	cFluor YG584	SK3	R7-20041-100T	Cytek612889
CD25	PE-CF594	M-A251	562403	BD
HLA-DR	AF647	L243	307622	BioLegend
CD19	Spark NIR 685	HIB19	302270	BioLegend
CD127	R718	HIL-7R-M21	352753	BD
CD27	APC-H7	M-T271	560222	BD
CD38	APC-Fire810	HIT2	356643	BioLegend

Figure S8 - Molecular biology differences between technologies

Examination of the different molecular biology features of the technologies tested in the PBMC samples in this study. A) Transcript metagene plot showing depth of coverage from transcript start (TSS) to transcript end (TES) for all expressed transcripts from each technology. The 10x 3pGEX align near the TES while HIVE and Evercode data are more uniformly distributed across the entire transcript length. The 10x FLEX data is not included in this plot because these data are derived from probes and are not compatible with whole transcript coverage plots. B) Expression levels of the example immune gene CD74 in each technology. Normalized and log10-transformed data from the data slot of the SCT assay are being presented. C) Sequence alignment data visualized using IGV in the CD74 example locus from each technology. The 10x

bioRxiv preprint doi: https://doi.org/10.1101/2025.10.24.684427; this version posted October 24, 2025. The copyright holder for this preprint (which was not certified by peer review) is the author/funder, who has granted bioRxiv a license to display the preprint in perpetuity. It is made available under aCC-BY 4.0 International license.

3p DGE reads mostly localize to the 3' end of the negative-stranded CD74 gene. The 10x FLEX alignments are restricted to the location of the FLEX probes (Red Arrowheads). Note that the middle probe is split between two exons and this results in a corresponding pair of read stacks. The HIVE and Evercode alignments are observed across the length of the transcript, predominantly over exons. Non-exonic alignments are often near genomic regions that are rich in A or T suggesting spurious genomic priming (data not shown).

Figure S1

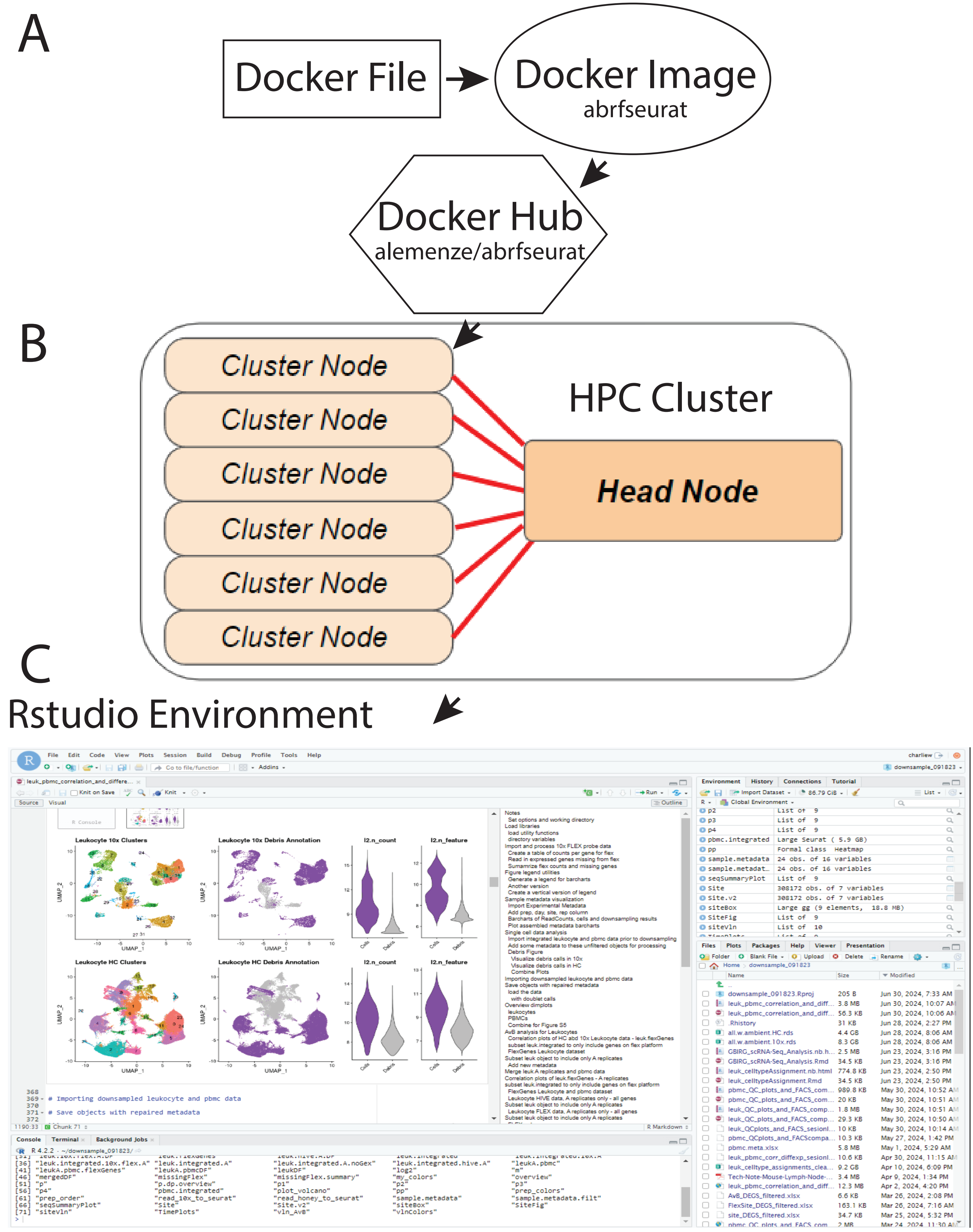
A
Docker File
Docker Image
abrfseurat
Docker Hub
alemenze/abrfseurat
B
Cluster Node
Cluster Node
Cluster Node
Cluster Node
Cluster Node
Cluster Node
HPC Cluster
Head Node
C
Rstudio Environment
Leukocyte 10x Clusters
Leukocyte 10x Debris Annotation
Leukocyte HC Clusters
Leukocyte HC Debris Annotation
Importing downsampled leukocyte and pbmc data
Save objects with repaired metadata

Figure S2

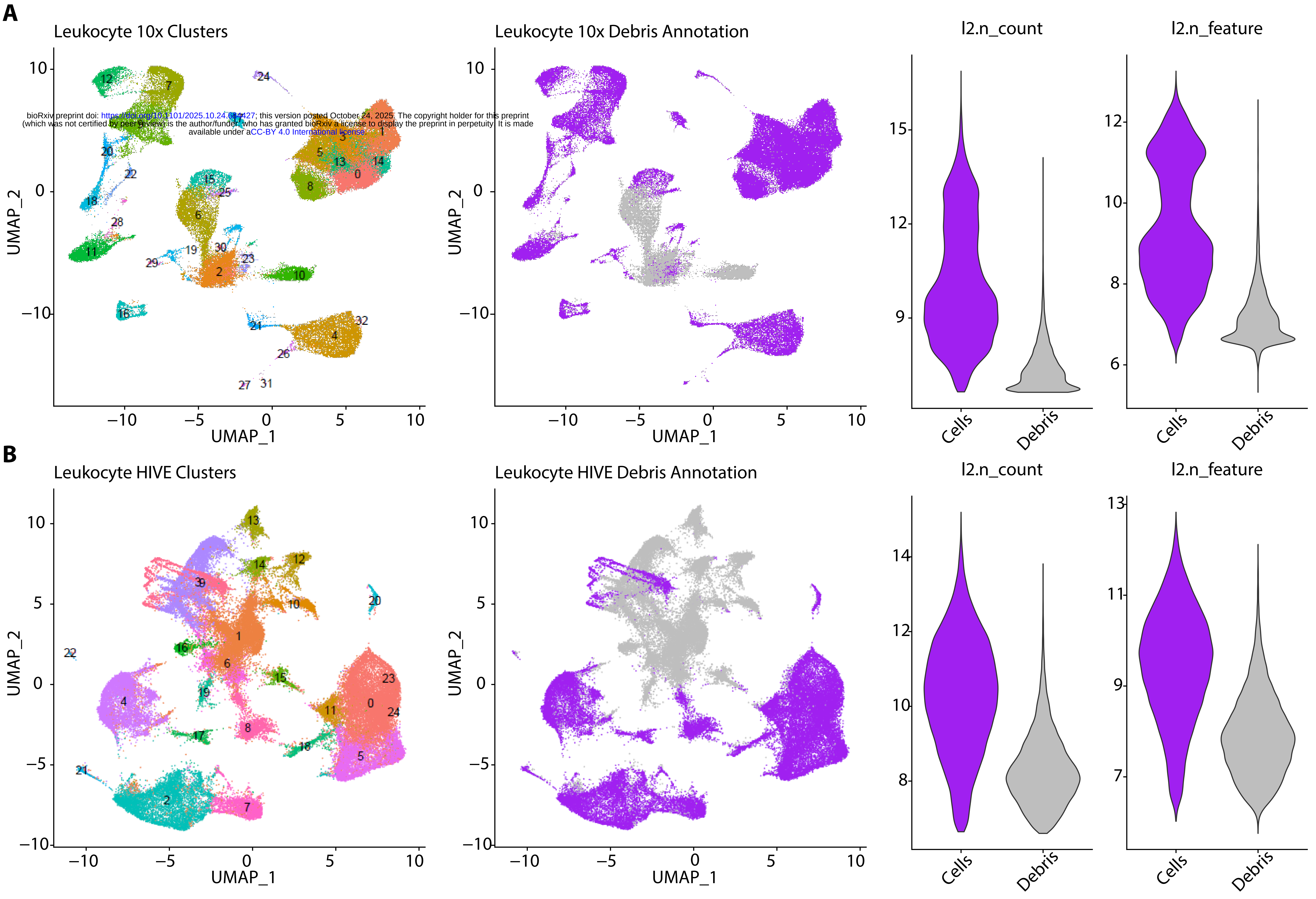

bioRxiv preprint doi: https://doi.org/10.1101/2025.10.24.684427; this version posted October 24, 2025. The copyright holder for this preprint (which was not certified by peer review) is the author/funder, who has granted bioRxiv a license to display the preprint in perpetuity. It is made available under aCC-BY 4.0 International license.

Figure S3

bioRxiv preprint doi: https://doi.org/10.1101/2025.10.24.684427; this version posted October 24, 2025. The copyright holder for this preprint (which was not certified by peer review) is the author/funder, who has granted bioRxiv a license to display the preprint in perpetuity. It is made available under aCC-BY 4.0 International license.

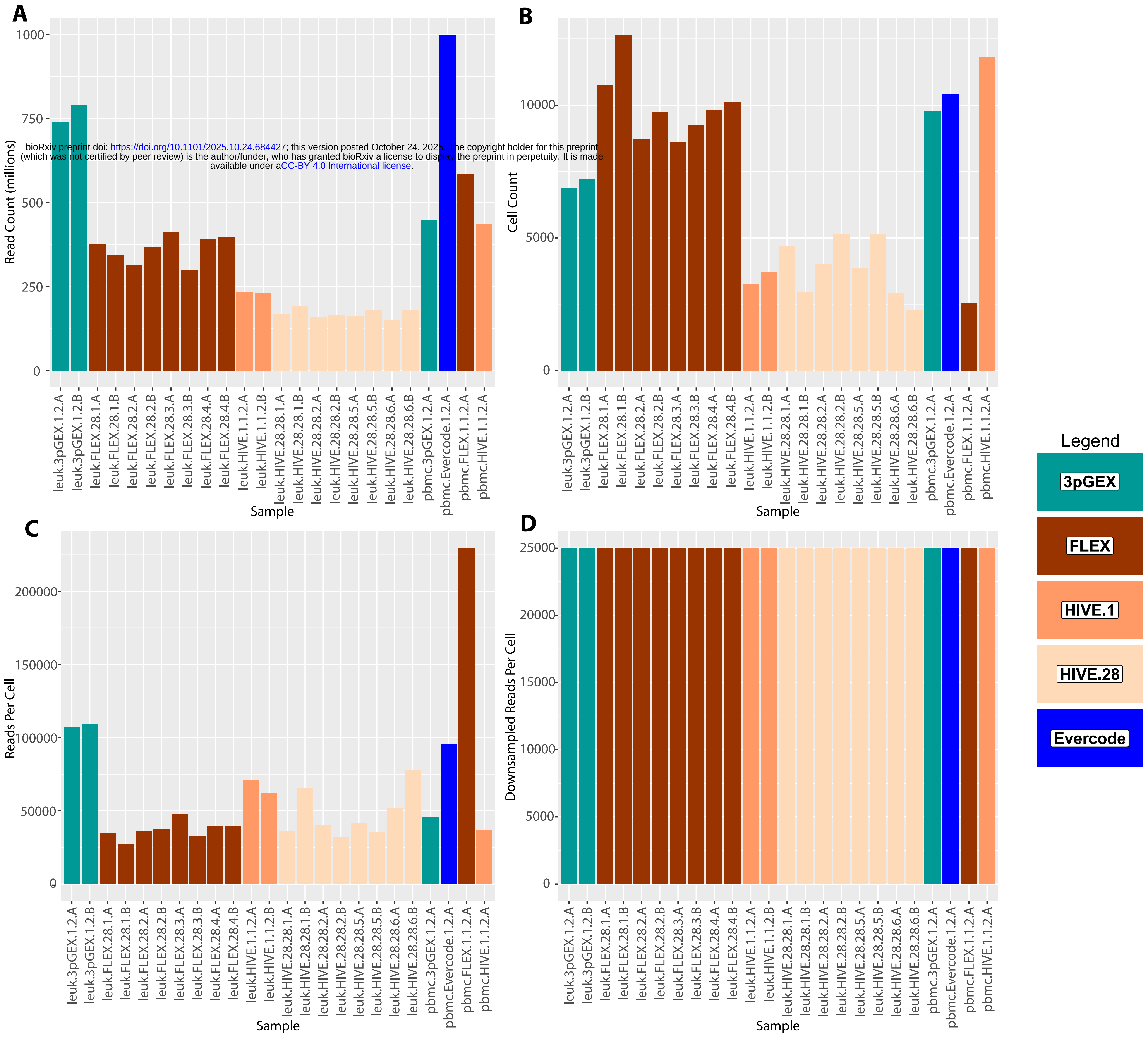

Figure S4

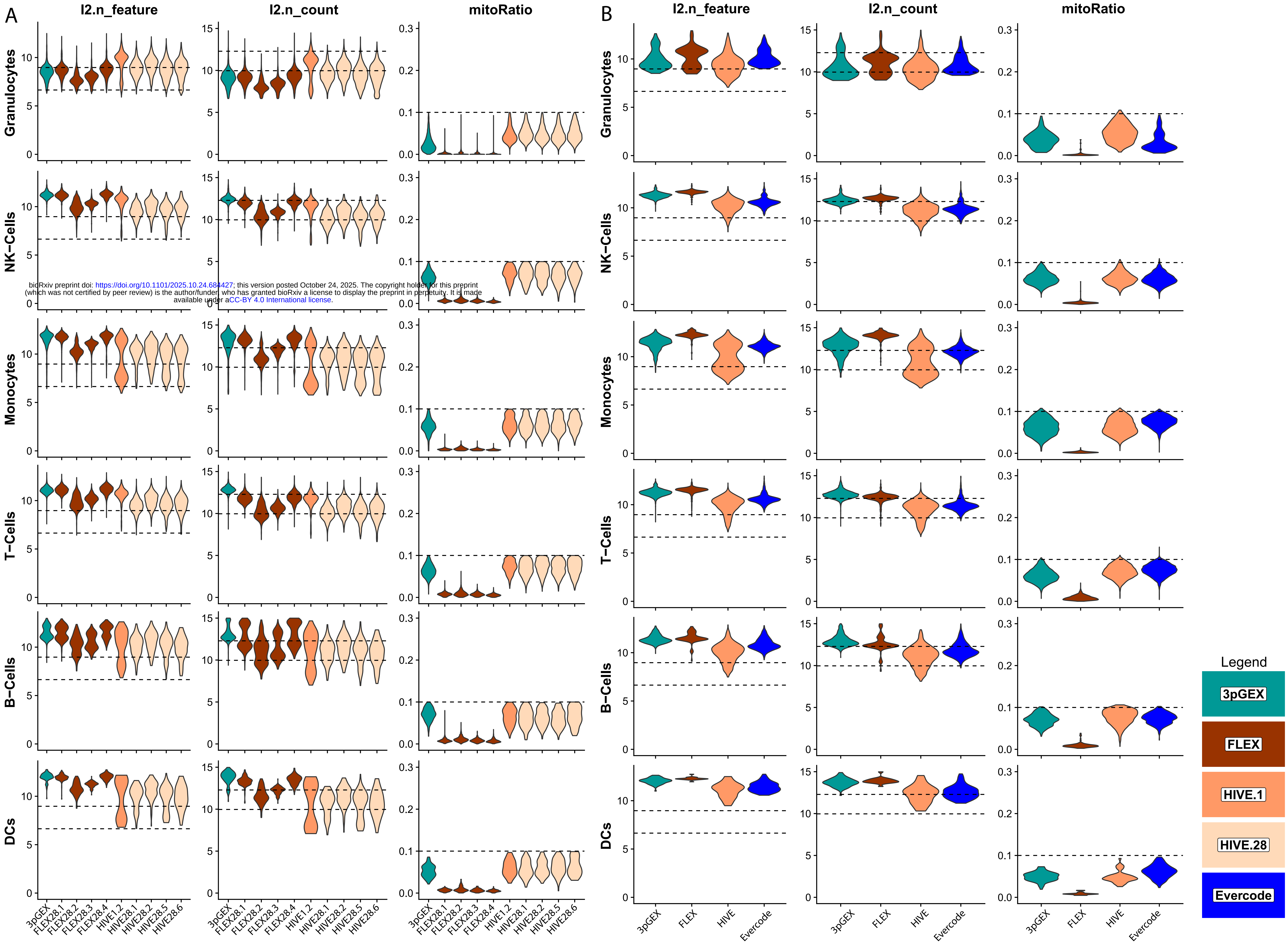

bioRxiv preprint doi: https://doi.org/10.1101/2025.10.24.684427; this version posted October 24, 2025. The copyright holder for this preprint (which was not certified by peer review) is the author/funder, who has granted bioRxiv a license to display the preprint in perpetuity. It is made available under aCC-BY 4.0 International license.

Figure S5

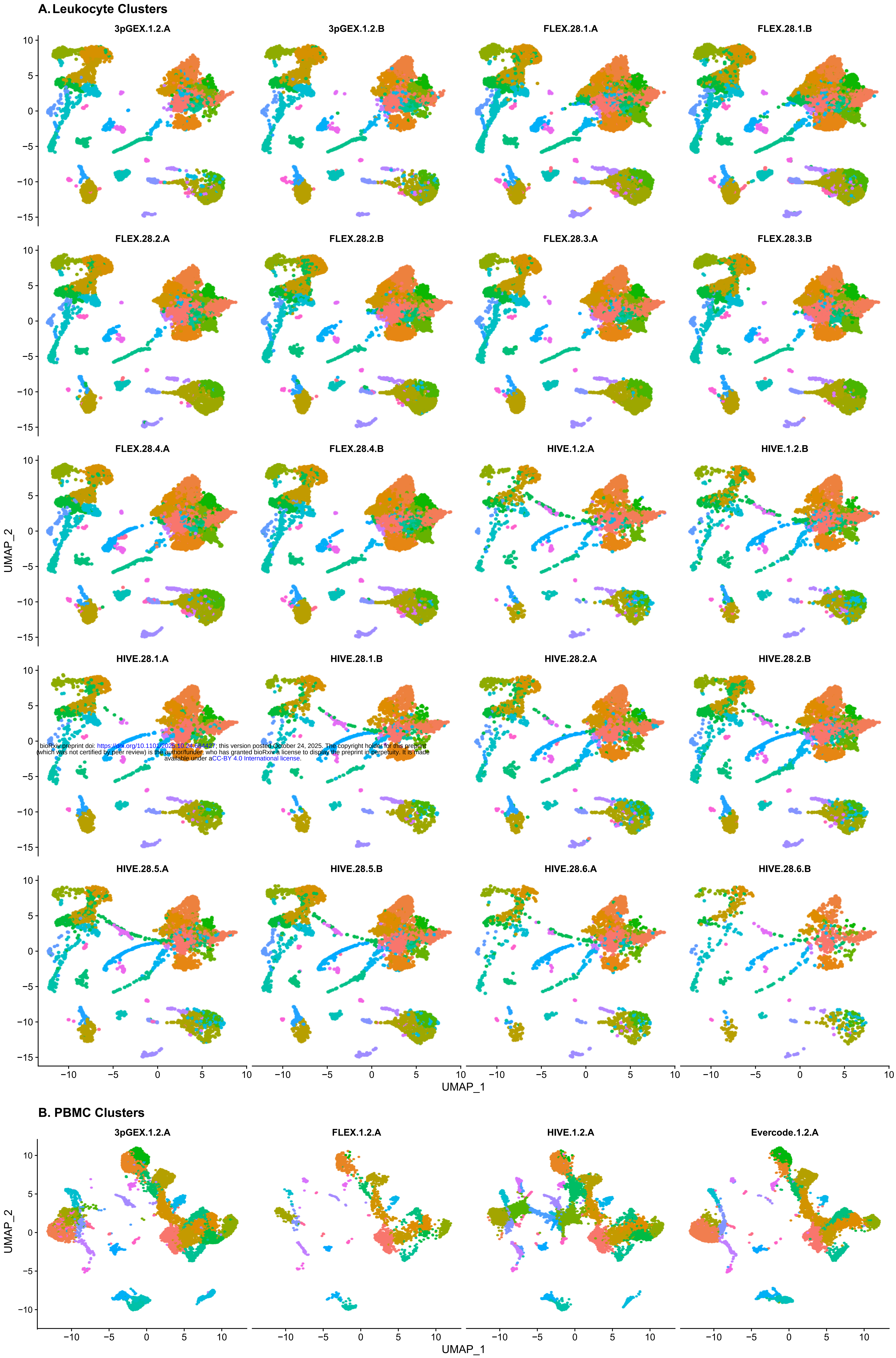

bioRxiv preprint doi: https://doi.org/10.1101/2025.10.24.684427; this version posted October 24, 2025. The copyright holder for this preprint (which was not certified by peer review) is the author/funder, who has granted bioRxiv a license to display the preprint in perpetuity. It is made available under aCC-BY 4.0 International license.

Figure S6

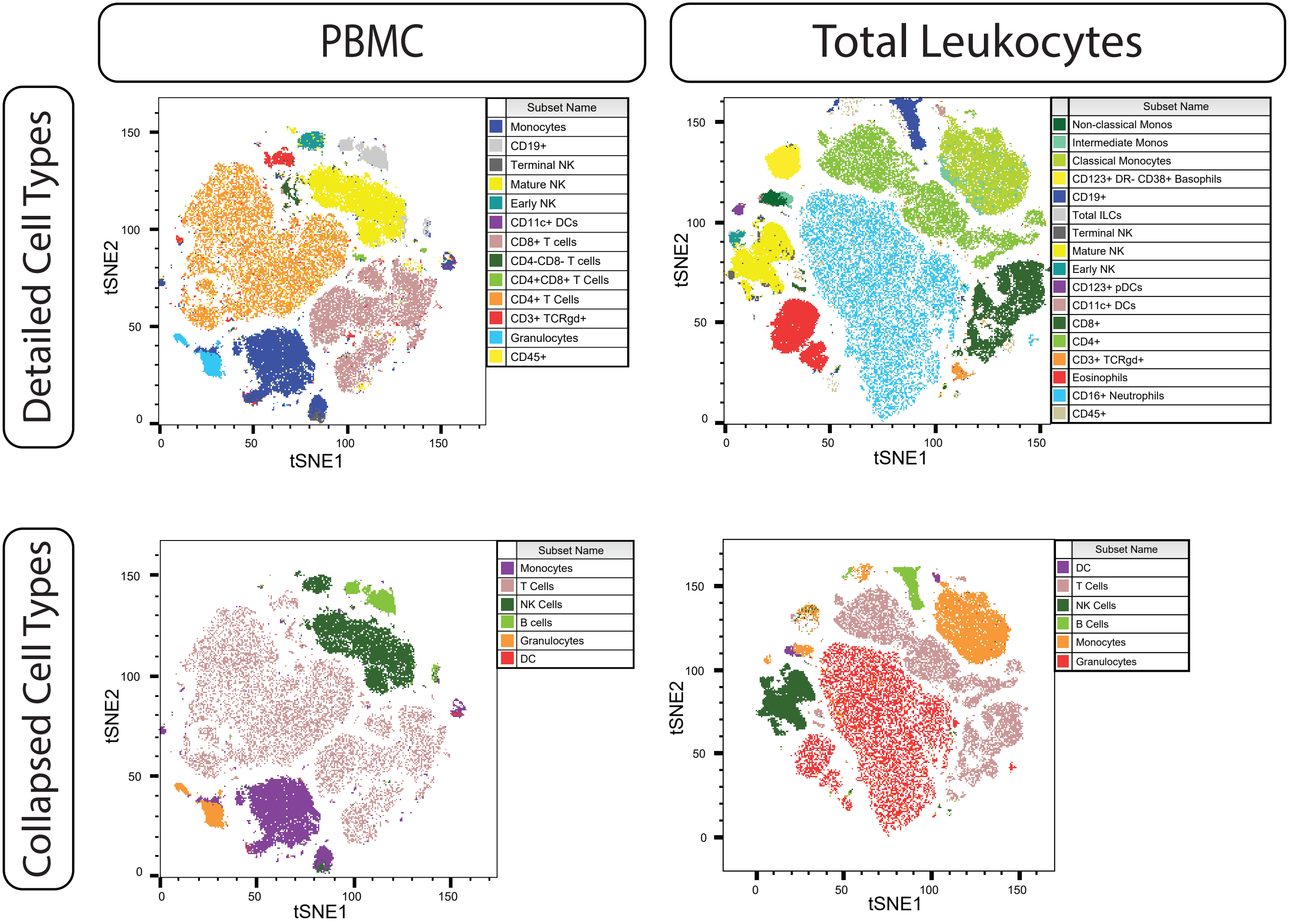
PBMC
Total Leukocytes
Detailed Cell Types
Collapsed Cell Types
tSNE1
tSNE2
Subset Name
Monocytes
CD19+
Terminal NK
Mature NK
Early NK
CD11c+ DCs
CD8+ T cells
CD4-CD8- T cells
CD4+CD8+ T Cells
CD4+ T Cells
CD3+ TCRgd+
Granulocytes
CD45+
Non-classical Monos
Intermediate Monos
Classical Monocytes
CD123+ DR- CD38+ Basophils
CD19+
Total ILCs
Terminal NK
Mature NK
Early NK
CD123+ pDCs
CD11c+ DCs
CD8+
CD4+
CD3+ TCRgd+
Eosinophils
CD16+ Neutrophils
CD45+
Monocytes
T Cells
NK Cells
B cells
Granulocytes
DC
DC
T Cells
NK Cells
B Cells
Monocytes
Granulocytes

Figure S7

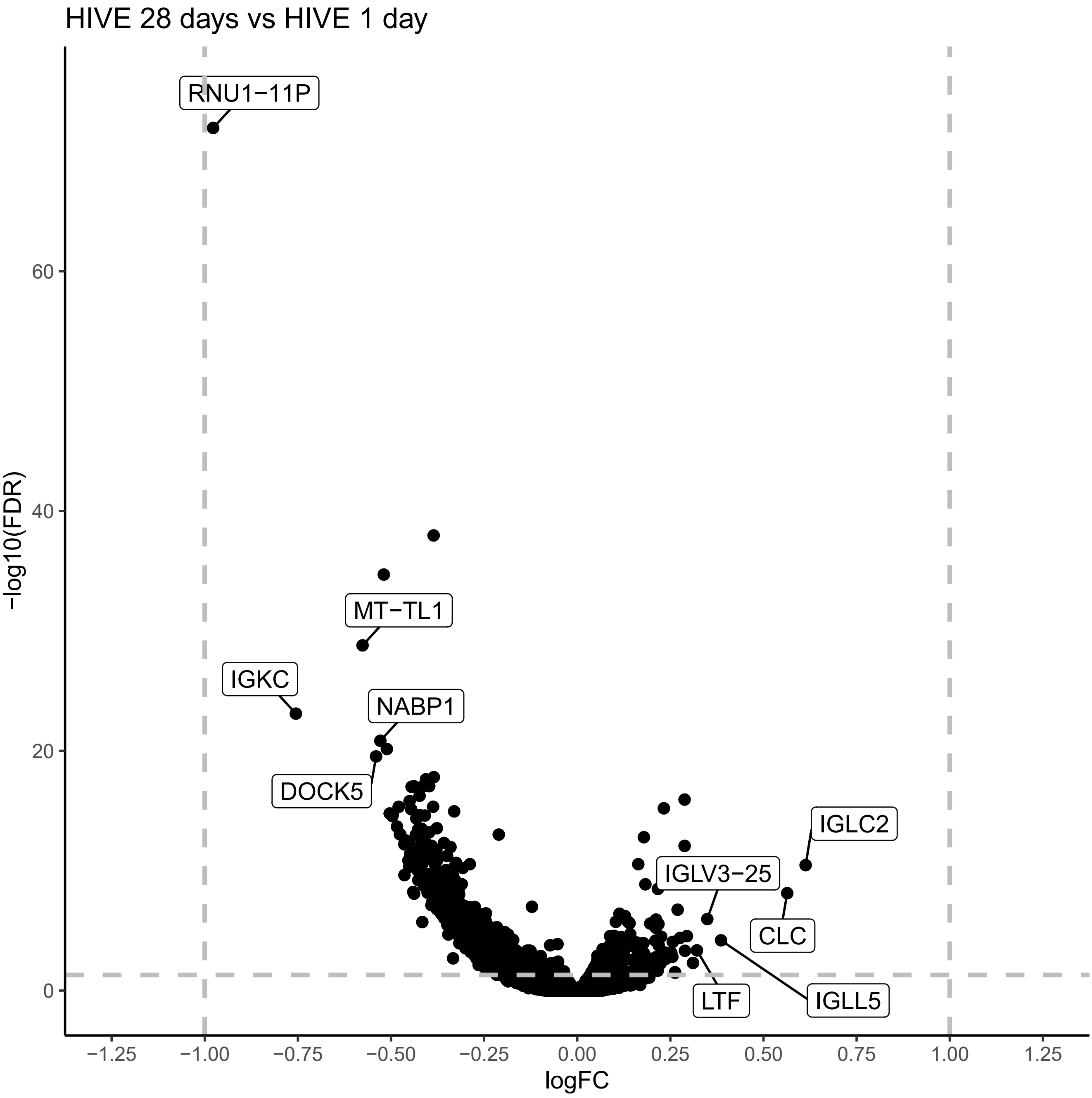
HIVE 28 days vs HIVE 1 day
RNU1−11P
MT−TL1
IGKC
NABP1
DOCK5
IGLC2
IGLV3−25
CLC
LTF
IGLL5
-log10(FDR)
60
40
20
0
−1.25
−1.00
−0.75
−0.50
−0.25
0.00
0.25
0.50
0.75
1.00
1.25
logFC

Figure S8

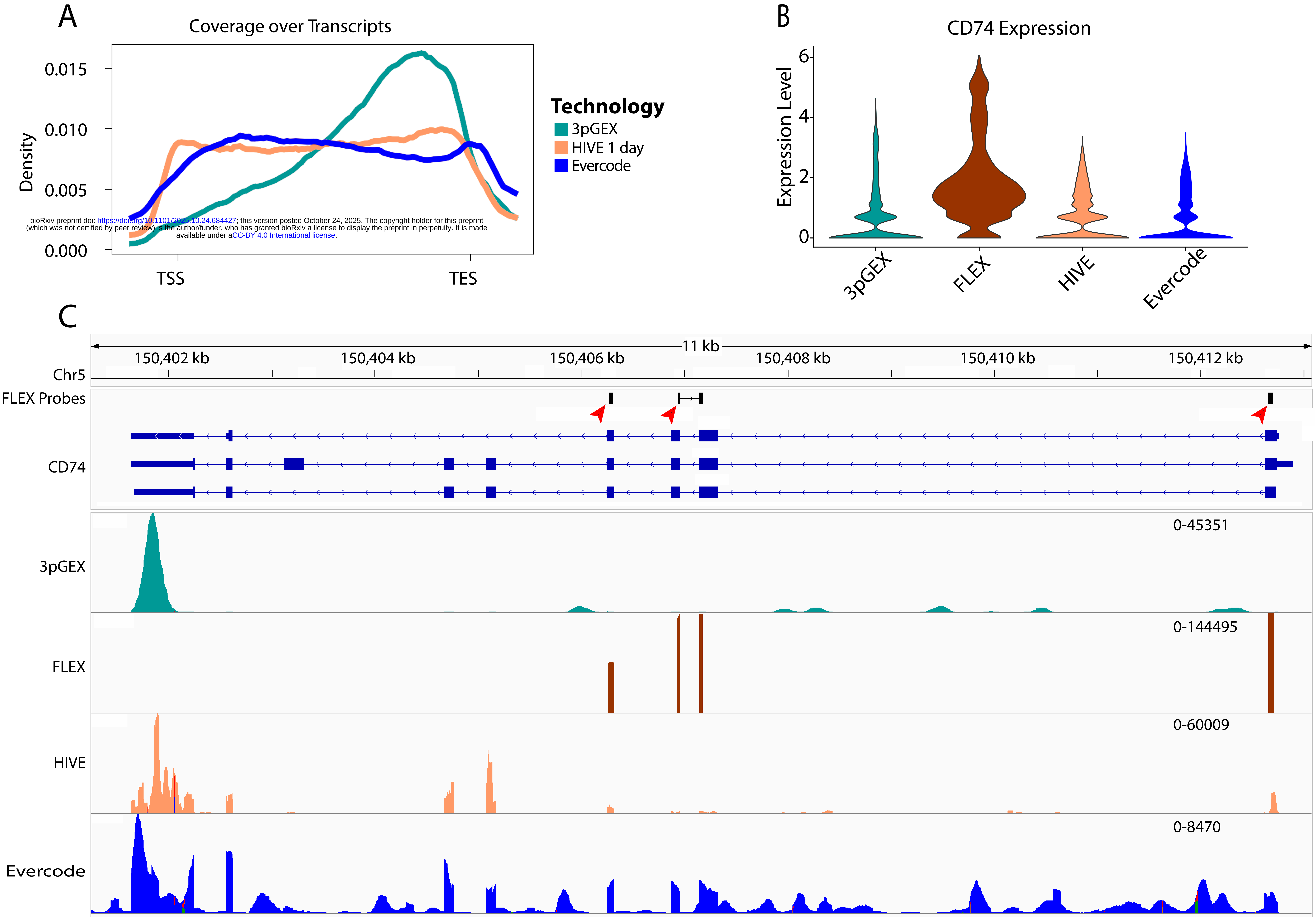

bioRxiv preprint doi: https://doi.org/10.1101/2025.10.24.684427; this version posted October 24, 2025. The copyright holder for this preprint (which was not certified by peer review) is the author/funder, who has granted bioRxiv a license to display the preprint in perpetuity. It is made available under aCC-BY 4.0 International license.
